# Implementation of an active aging model in Mexico for prevention and control of chronic diseases in the elderly

**DOI:** 10.1186/1471-2318-9-40

**Published:** 2009-08-26

**Authors:** Víctor Manuel Mendoza-Núñez, María de la Luz Martínez-Maldonado, Elsa Correa-Muñoz

**Affiliations:** 1 Unidad de Investigación en Gerontología, Facultad de Estudios Superiores Zaragoza, Universidad Nacional Autónoma de México (UNAM), México D.F., México

## Abstract

**Background:**

World Health Organization cites among the main challenges of populational aging the dual disease burden: the greater risk of disability, and the need for care. In this sense, the most frequent chronic diseases during old age worldwide are high blood pressure, type 2 diabetes mellitus, cancer, arthritis, osteoporosis, depression, and dementia. Chronic disease-associated dependency represents an onerous sanitary and financial burden for the older adult, the family, and the health care system. Thus, it is necessary to propose community-level models for chronic disease prevention and control in old age. The aim of the present work is to show our experience in the development and implementation of a model for chronic disease prevention and control in old age at the community level under the active aging paradigm.

**Methods/Design:**

A longitudinal study will be carried out in a sample of 400 elderly urban and rural-dwelling individuals residing in Hidalgo State, Mexico during five years. All participants will be enrolled in the model active aging. This establishes the formation of 40 gerontological promoters (GPs) from among the older adults themselves. The GPs function as mutual-help group coordinators (gerontological nuclei) and establish self-care and self-promotion actions for elderly well-being and social development. It will be conformed a big-net of social network of 40 mutual-help groups of ten elderly adults each one, in which self-care is a daily practice for chronic disease prevention and control, as well as for achieving maximal well-being and life quality in old age. Indicators of the model's impact will be (i) therapeutic adherence; (ii) the incidence of the main chronic diseases in old age; (iii) life expectancy without chronic diseases at 60 years of age; (iv) disability adjusted life years lost; (v) years of life lost due to premature mortality, and (vi) years lived with disability.

**Discussion:**

We propose that the implementation of the model active aging framework will permits the empowerment of older adults, which constitutes basic social capital for chronic disease prevention and control in old age.

## Background

The challenges of populational aging involve everyone. In this regard, the United Nations reported that in 2007 there were 700 million persons aged 60 years and over on the planet, which represents 11% of the entire world population. Likewise, it is projected that this percentage will increase to 15% by the year 2025 and to 22% by 2050 [1]. In Mexico in the year 2005, it was informed that there were 8.4 million individuals aged 60 years and over (8.1% of the total population), and it has been projected that that there will be 17.5 million (12.4%) by 2025 and 35.7 million (24.3%) by 2050 [2].

On the other hand, the World Health Organization (WHO) cites among the main challenges of populational aging the dual disease burden: the greater risk of disability, and the need for care [3].

The most frequent chronic diseases during old age worldwide are high blood pressure, type 2 diabetes mellitus, cancer, arthritis, osteoporosis, depression, and dementia [3,4], In this regard, in Mexico high blood pressure presents in the 50% and type 2 diabetes mellitus, in the 20% of adults aged >60 years [5,6].

We observed the principal repercussions of chronic diseases in old age in terms of physical, mental, and social functionality, affecting basic, instrumental, and advanced activities of daily life. Chronic disease-associated dependency represents an onerous sanitary and financial burden for the older adult, the family, and the health care system [7,8].

In the Second World Assembly on Ageing held in Madrid in 2002, the relevance of active aging was highlighted as a key strategy for achieving the maximum health, well-being, and quality of life (QOL) of older adults, defining this as "the process of optimizing opportunities for health, participation and security in order to enhance quality of life as people age" [4].

**Figure 1:**
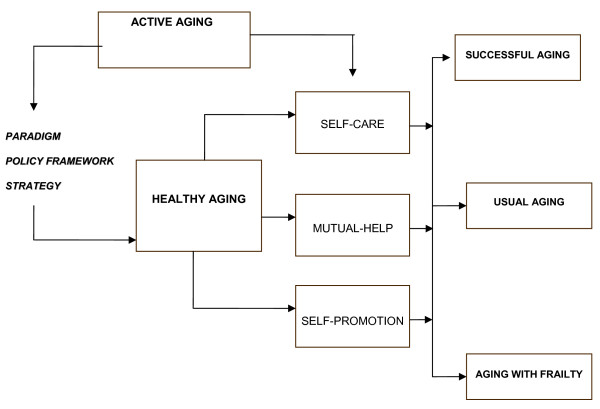
**Active aging as paradigm, policy framework, and strategy for healthy aging**.

Active aging refers to the empowerment of older persons in biological, psychological, and social areas, understanding empowerment as the individual's self-promotion, independence, and self-confidence, as well as his/her right to a dignified way of life according to self-imposed values, the ability to stand up for one's own rights, and to be free [4]. Active aging entertains three levels of approach, including a) paradigm, b) policy strategy, and c) instrumental action at the community level (Figure 1).

**Figure 2:**
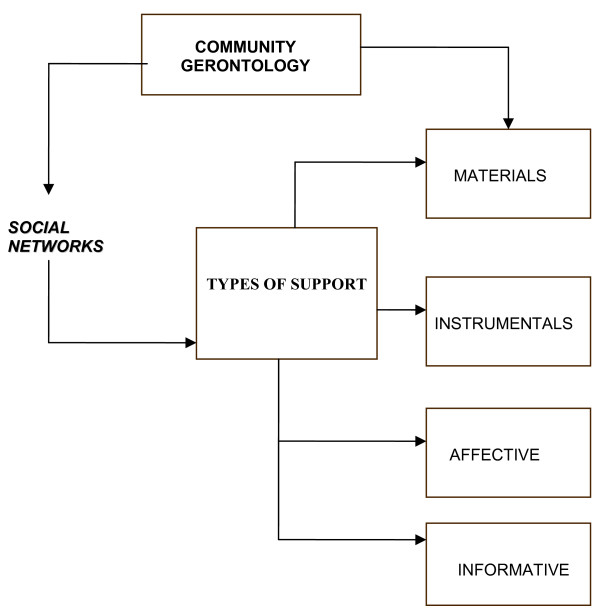
**Types of support that elderly individuals can offer through social networks of social support in Community Gerontology programs**.

Social networks refer to the personal, community, and institutional contacts by means of which the individual maintains his/her social identity and receive material, instrumental, emotional, and informative support (Figure 2). In these terms, social capital depends to a great extent on the social contacts that the individual possesses [9-11], thus the importance of generating and strengthening older adults' social networks in formal programs with specific objectives and goals.

The type of social support that social networks can afford are the material, instrumental, affective, and informative provisions, real or perceived, provided by family, friends, the community, and formal institutions [9-11].

Our research group developed an active aging model for chronic disease prevention and control in old age at the community level, comprising a big-net of social networks of mutual-help of elders in which self-care is a daily practice [12,13].

Thus, it is necessary to propose community-level models for chronic disease prevention and control in old age. The aim of the development and implementation of a model active aging is reach the empowerment of elderly-dwelling individuals for chronic disease prevention and control in old age at the community through self-care, self-help and self-promotion.

## Methods/design

### Design and subjects

A longitudinal study will be carried out in a sample of 400 elderly urban and rural-dwelling individuals residing in Hidalgo State, Mexico during five years, with a significance level of 0.05, a power of 80% and an expected difference in chronic diseases incidence of 50% [14] between the pre-intervention and post-intervention. Taking into account a drop-out rate of 30% a minimum of 280 participants are needed in the cohort.

The subjects agreed to participate in the study after giving their informed consent. The Ethics Committee of the Universidad Nacional Autónoma de México (UNAM) Zaragoza Campus approved the research protocol for this study.

All participants will be enrolled in the model active aging. This establishes the formation of 40 gerontological promoters (GPs) from among the older adults themselves. The GPs function as mutual-help group coordinators (gerontological nuclei) and establish self-care and self-promotion actions for elderly well-being and social development. It will be conformed a big-net of social network of 40 mutual-help groups of ten elderly adults each one, which will be established actions of self-care as daily practice for chronic disease prevention and control, as well as for achieving maximal well-being and life quality in old age.

### Characteristics of the Model

As a key element, the model establishes the training of gerontological promoters (GPs) from among the older adults themselves. The GPs function as mutual-help group coordinators (gerontological nuclei) and establish self-care and self-promotion actions for elderly well-being and social development.

Self-care at the gerontological level refers to the reasoned theoretically based behavior of the individual that allows the elderly adult to decide and act upon the prevention, diagnosis, and treatment of his/her disease, as well as the maintenance of health and maximum enjoyment of QOL, according to his/her sociocultural context, utilizing formal and informal social networks in optimal fashion during aging. Similarly, the mutual-help including reasoned solidarity-oriented behavior that is adopted by a group of elderly individuals who share like problems and whose members are aware of the advantages and commitments acquired on accepting voluntarily to form part of the group. Concerning self-promotion, this involves the actions that an older adult or a self-help group carries out autonomously, in advance, and in optimal form and considers elements and mechanisms of formal and informal social support networks.

**Figure 3:**
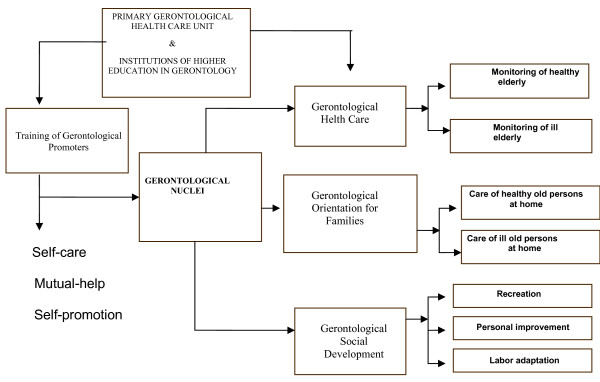
**Gerontological care model**. Primary Gerontological Health Care Unit linked to Institutions of Higher Education in Gerontology is responsible for the design and implementation of educational programs and guides the training of the gerontological promoters for the development of mutual-help groups (gerontological nucleus). A gerontological nuclei is a group integrated by 10 to 15 older adults of nearby communities with similar interests. They are mainly involved in the practice of self-care, mutal-help, and self-promotion guidelines established by the program. The model is addressed toward the following objectives: (i) the supervision of the gerontological health status of participants in the program; (ii) the training of qualified gerontological health care promoters, certified on the basis of a formal continuous education program offered by a renowned academic institution; (iii) to provide orientation and guidance to families with regard to basic care practices with both healthy and sick older adults; (iv) to promote the social and gerontological development of the older populations in Mexico.

The model contemplates a Primary Gerontological Health Care Unit (PGHCU), whose purpose is to coordinate the big-net of social networks of mutual-help groups of elderly adults, in which self-care is a daily practice for chronic disease prevention and control, as well as for achieving maximal well-being and QOL in old age (Figure 3).

The PGHCU linked with Institutions of Higher Education in Gerontology will be responsible for the training of gerontological promoters (GP) following the formal academic structure of a workshop (Table 1) and considering the following principles for teaching older adults [15,16]: (i) *Mature individuals learn only what they want to learn; *(ii)*Mature individuals learn only what they are capable of learning; *(iii) *Mature individuals learn mainly what they teach themselves, and *(iv)*Mature individuals learn mainly in terms of their experience*.

**Table 1:** Workshop community gerontology topics

Age, aging and aged	Gender and aging	Nails care in the elderly
Second World Assembly on Aging	Sexuality in older adults	Foots care in the elderly
Active aging	Accidents in the elderly	Skin care in the elderly
Successful aging	Chronic diseases in the aging	Sleep hygiene in the elderly
Healthy aging and functionality	Prevention of diseases	Physical exercise and aging
Empowerment in the aging	Diabetes mellitus	Mouth and teeth care in the elderly
Gerontological promoters	Arterial hypertension	Dental prosthesis care in the aging
Self-care, mutual-help and self-promotion	Mild cognitive impairment	Social-support nets
Gerontological care model	Depression	Thanatology
Age-related biological changes	Cancer	Leisure and aging
Age-related psychological changes	Osteoporosis	Self-esteem and aging
Age-related social changes	Polypharmacy	Laws and aging
Ageism	Vaccination in the aging	Abuse and aging
The elderly and their families	Nutrition in the aging	Life quality and aging

The requirements for participating in training courses for GPs are as follows: (i) interest in participating in an intensive training program focused on holistic gerontological development; (ii) 60-74 years of age; (iii) literate; (iv) absence of handicapping illnesses or serious visual or auditory disabilities, and (v) leadership attributes and the ability to coordinate small groups.

We will implement a 60-hour workshop that integrated both theoretical and practical aspects (14 weekly sessions). An introductory textbook entitled "Community Gerontology" was designed specifically for this aim [17].

Topics for the workshop were selected and approved by a panel of four Gerontologists and took into consideration their basic knowledge on Community Gerontology and age-related changes in the following: biological; psychological, and social aspects; prevention of chronic diseases; healthy lifestyle in the aging period; empowerment, and social networks (Table 1), according to the active aging paradigm.

The pillars of the model are as follows: gerontological health care; gerontological orientation for families, and gerontological social development.

#### Gerontological Health Care (GHC)

**Figure 4:**
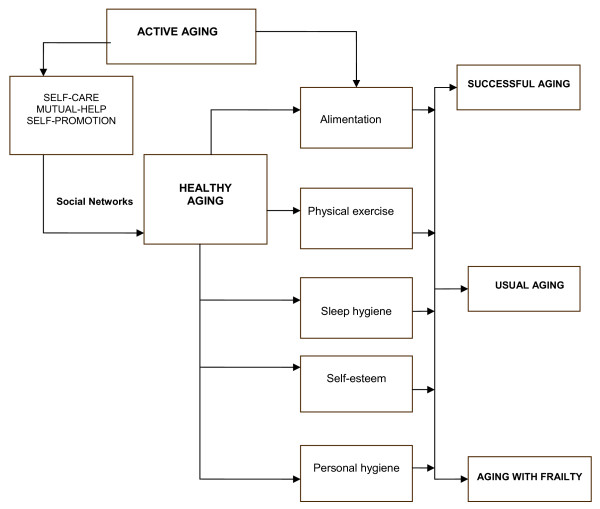
**Lifestyles for healthy aging for elderly individuals with successful, usual, and fragile aging**.

The fundamental objective of GHC is to prevent and detect the diseases of greatest prevalence in the elderly, such as high blood pressure, diabetes mellitus, depression, osteoarthrosis, cognitive deterioration, and osteoporosis, as well as establishing actions of healthy aging for the maintenance, prolongation, and recuperation of physical, mental, and social functionality (Figure 4). The active aging program also seeks to improve the self-perception of psychosocial well-being, considering the elderly adult's physical condition and sociocultural environment. Thus, control programs should be implemented for the healthy and the ill older adult, with pre-established evaluation, surveillance, and primary health care actions. These actions should be performed by previously trained older adults who are coordinated by GPs, who are in turn supported and supervised by the PGHCU.

#### Gerontological Orientation for Families (GOF)

GPs should possess sufficient knowledge for orienting and training family members to provide basic care for the healthy and ill elderly adult in the home, with the purpose of preventing diseases and/or their complications, in addition to promoting healthy lifestyles for the maintenance, prolongation and recuperation of physical, mental, and social functionality.

#### Gerontological Social Development (GSD)

Among its goals, the model contemplates maximum enjoyment by older adults of their situation of being old. Thus, implantation is recommended of programs of recreation, adaptation, and psychosocial and occupational excelling within an anthropological focus, according to the elderly individual's interests, age, schooling, gender, health state, and socioeconomic situation.

The model established flexible general guidelines framed within an active aging paradigm. Therefore, actions for GSD should be adopted by the population in which the model is implemented (rural or urban), as well as by older adult groups of different sociocultural and economic conditions.

### Model Implementation

In 2007, an agreement was signed between the National Autonomous University of Mexico (UNAM) and the Institute for Care of the Elderly in the State of Hidalgo (Mexico) with the objective of implementing a State Active Aging Program according to the model developed at the UNAM (Zaragoza Campus).

Among actions implemented within the agreement's framework, we are able to highlight the following: (i) the training of professional personnel of the Institute on the active aging paradigm; (ii) training of gerontological promoters; (iii) the editing of a text on Community Gerontology direct toward elderly adults, and (iv) in 2008, the Institute adopted the Active Aging paradigm as public and strategic policy for care of the elderly.

Indicators of the model's impact will be the following: (i) empowerment; (ii) therapeutic adherence; (iii) the incidence of the main chronic diseases in old age; (iv) life expectancy without chronic diseases at 60 years of age; (v) disability adjusted life years lost (DALYs); (vi) years of life lost due to premature mortality (YLL), and (vii) years lived with disability (YLD).

## Discussion

Walter (2006) proposes the following seven principles that allow for delineating the components of active aging: (i) participation should contribute to the well-being of the older adult; (ii) it should have a preventive focus; (iii) it should be within the reach of the entire population of elderly individuals, including those who are frailty and dependent, with the degree of participation according to the physical, psychological, and social conditions of the older adult; (iv) it should propitiate the maintenance and strengthening of intergenerational solidarity; (v) it should take into consideration the person's rights and obligations; (vi) it should be participative with empowerment, and (vii) it should contemplate national, local elements and cultural diversity [18].

The following are the pillars of active aging are health, participation, and security: (i) the prevention and control of chronic diseases, as well as maintaining, prolonging, and recuperating physical, mental, and social functionality according to the older adult's age and specific sociocultural context should be considered as fundamental elements with regard to health; (ii) concerning participation, opportunities should be provided that permit older adults to have productive social participation in remunerated and unremunerated activities, in programs linked with social development, work, education, health, cultural and spiritual development, etc., according to elderly persons' rights, capabilities, needs, and preferences; (iii) concerning security, policies should be established that guarantee permanent access to health, alimentation, housing, and well-being for functional older adults, as well as for those requiring instrumental care.

On the other hand, active aging as a policy framework constitutes a feasible alternative for substituting the current model, which visualizes the elderly adult as a sickly being and one in decadence, without the possibility of economic and social development. Therefore, the elderly are frequently considered a social and economic burden.

The development of community care models within the framework of active aging is a call and a challenge to which we should devote ourselves in all countries. In this regard, we should confront the present hegemonic view of aging within the framework of a structured dependency, which has propitiated a marketing focus in parallel fashion on covering hospital and care needs, developing in parallel great industries of technical, care, and drug industries for aging, which indirectly has negative repercussions on physical, mental, and social functionality [19]. This focus promotes and links well-being, care, and affect for the elderly with these aids, which are frequently unnecessary.

On the other hand, there is an ever greater demand for health care for older adults, outpacing in some cases the resources allocated for this purpose. Thus, there is a need to propose alternative care models that exert a significant impact on cost-benefit and on the QOL of the elderly [20]. In this regard, it has been demonstrated at the community level that social support and education are fundamental for chronic disease control accompanied by a significant impact on cost-benefit [21].

**Figure 5:**
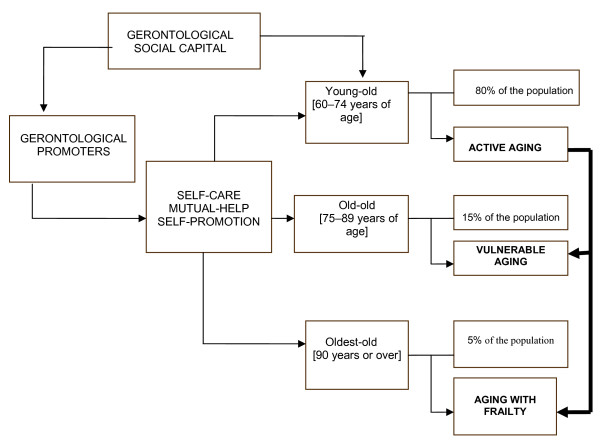
**Gerontological social capital and active aging**.

In different studies, the social capital has been recognized of the group of young-old individuals (aged 60-74 years) for active aging, because >80% of this age group are functional and can potentially provide material, instrumental, affective, and informative support to other older adults [22-24] (Figure 5).

## Conclusion

We propose that the implementation of the model active aging will permit us to reach the empowerment of older adults for self-care, and consequently prevent and control the diseases chronic, besides its complications.

## Competing interests

The authors declare that they have no competing interests.

## Authors' contributions

VMMN conceived and designed the study, drafted the manuscript. MLMM participated in the design of the study and interviews of the participants. ECM participated in the design of the study and interviews of the participants. All authors read and approved the final manuscript.

## Pre-publication history

The pre-publication history for this paper can be accessed here:


